# Supplementary material for: Causal Relationship between Aging and Anorexia Nervosa: A White-Matter-Microstructure-Mediated Mendelian Randomization Analysis
Source: Biomedicines. 2024 Aug 16;12(8):1874. doi: 10.3390/biomedicines12081874 (PMC11351342; doi:10.3390/biomedicines12081874)
Supplement: Supplementary file 1 [file biomedicines-12-01874-s001.zip › Supplementary Figure/Figure S9/Leaveoneout plot of MR analysis for ubm-b-1720 to AN.pdf]

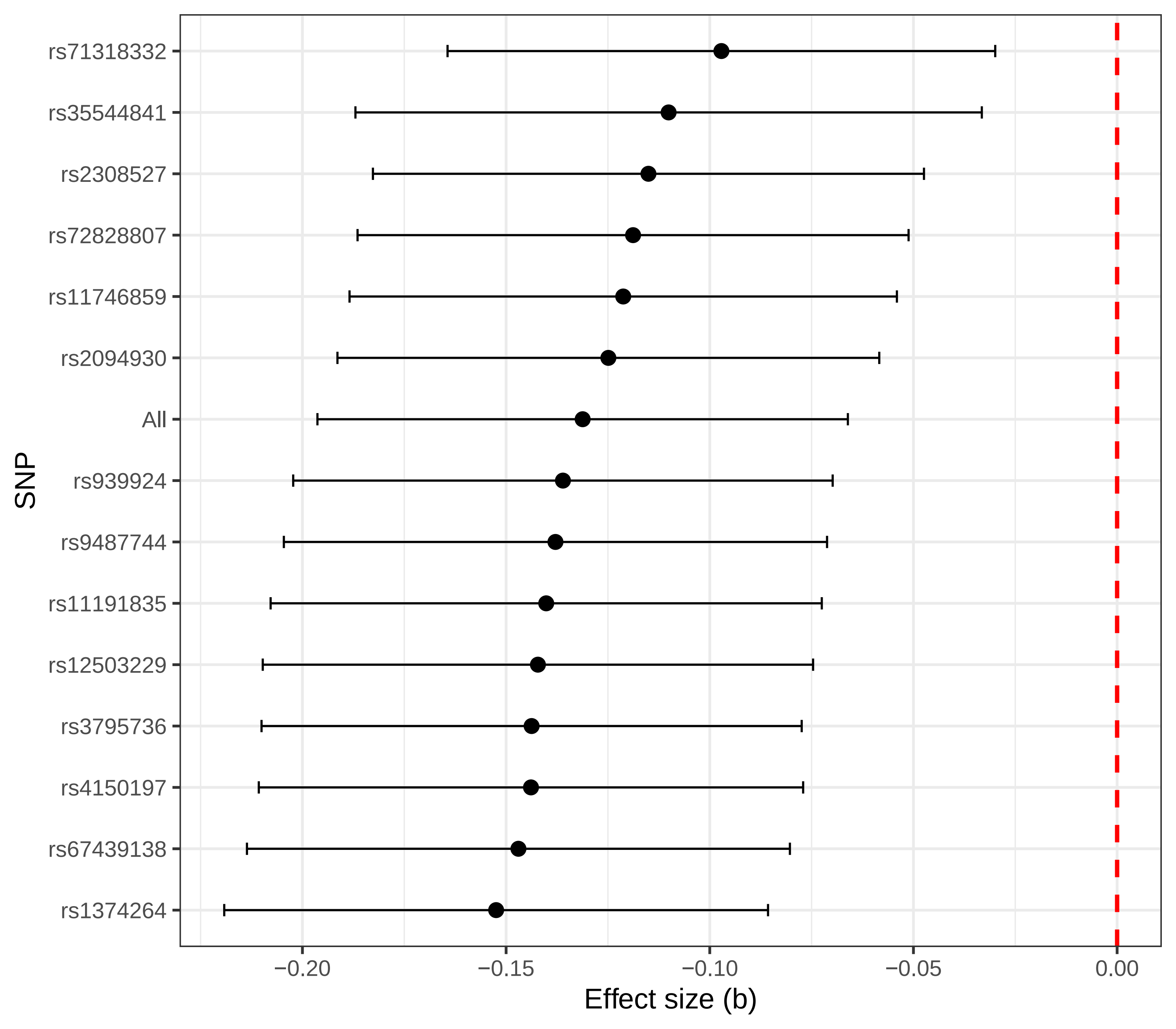
rs71318332
rs35544841
rs2308527
rs72828807
rs11746859
rs2094930
All
rs939924
rs9487744
rs11191835
rs12503229
rs3795736
rs4150197
rs67439138
rs1374264
SNP
−0.20
−0.15
−0.10
−0.05
0.00
Effect size (b)
